# Opinion analysis and aspect understanding during covid-19 pandemic using BERT-Bi-LSTM ensemble method

**DOI:** 10.1038/s41598-022-21604-7

**Published:** 2022-10-12

**Authors:** Mayur Wankhade, Annavarapu Chandra Sekhara Rao

**Affiliations:** Department of Computer Science and Engineering, Indian Institute of Technology (ISM), Dhanbad, Jharkhand 826004 India

**Keywords:** Computer science, Information technology, Health services

## Abstract

Social media platforms significantly increase general information about disease severity and inform preventive measures among community members. To identify public opinion through tweets on the subject of Covid-19 and investigate public sentiment in the country over the period. This article proposed a novel method for sentiment analysis of coronavirus-related tweets using bidirectional encoder representations from transformers (BERT) bi-directional long short-term memory (Bi-LSTM) ensemble learning model. The proposed approach consists of two stages. In the first stage, the BERT model gains the domain knowledge with Covid-19 data and fine-tunes with sentiment word dictionary. The second stage is the Bi-LSTM model, which is used to process the data in a bi-directional way with context sequence dependency preserving to process the data and classify the sentiment. Finally, the ensemble technique combines both models to classify the sentiment into positive and negative categories. The result obtained by the proposed method is better than the state-of-the-art methods. Moreover, the proposed model efficiently understands the public opinion on the Twitter platform, which can aid in formulating, monitoring and regulating public health policies during a pandemic.

## Introduction

With the emergence of Covid-19 globally massive number of people affected have suffered health issues, making the epidemic the most significant public health event of the twenty-first century. The impact of the disease has been a life-threatening a health condition that cannot be disregarded throughout previous years in history, and it has impacted people physiology and psychology. The Wuhan health commission in china recorded 27 instances of pneumonia with no known cause in December 2019. Covid-19, which is generated from SARS-CoV-2, is spreading rapidly worldwide, causing millions of infections and deaths among humans. SARS-CoV-2 was discovered in a seafood marketplace in China in December 2019 and infected millions of people. The World Health Organization (WHO) (https://www.who.int) formally named the novel coronavirus Covid-19 on 11 Feb 2020 in Geneva. Since the outbreak of Covid-19, the disease has sparked widespread panic worldwide.

The Covid-19 epidemic had a profound effect on social communities and people’s daily life throughout the world. Countries globally employed various containment guidelines in response to local pandemic conditions to safeguard their populations and restrict the spread of Covid-19. To enforce lockdown and suspend domestic and international flights to routine methods such as making it mandatory to wear masks and maintain social distancing in public. Countries have used various methods to control the spread of covid-19. While people in most countries have adapted to covid appropriate safety guidelines such as masks and social distancing, a complete restriction on movement enforced through a lockdown has often met with resistance in different parts of the world. The public perception of a lockdown is crucial since it determines public opinion. Real-time analysis of posts on social media has proven to be a valuable resource for understanding public sentiment. The method of identifying community opinion is known as “sentiment analysis”. It is a subset of text mining that entails automating emotions from textual content through natural language processing (NLP). Textual analytics is concerned with evocating and analyzing characters. Also, as the semantics, syntax, and associated endogenous aspects. This method aids in defining user opinion toward any entity, issue, or product, among other things ^1,2^.

Social media platforms facilitate information among human societies and assist them in exchanging ideas, information, knowledge, and other facts. It generates a massive volume of data on various platforms like Facebook, Twitter, and Instagram. Social and statistical research has revealed that regular usage of these applications affects human behavior, both positively and negatively. Twitter has approximately 400 million registered users sharing their thought and generates a massive data volume every day. Twitter users share news, information, and opinions through “ tweets,” short messages. Twitter users can also like, comment, “retweet” (reposting a tweet), and share tweets. This provides an excellent platform for opinion-based textual data analytics in various real-world applications. According to WHO guidelines, awareness and social isolation are the most effective methods of staying safe during a pandemic. Social distancing is an effective strategy to control the spread of the Covid-19 pandemic. People are encouraged to remain at home and maintain social distance. To ensure social separation in public spaces, practically all governments imposed lockdowns. There has never been a time when the entire planet was under lockdown in the history of human civilization. Lockdown is an emergency technique that prevents individuals from freely moving about public areas. People must stay there and not even venture outside their building during a total lockdown. Consider lockdown as a curfew with certain exceptions for vital services. For the duration of the closure, all non-essential services are disabled. Over 200 countries, regions, and territories have already been affected by the Covid-19 outbreak. The Covid-19 situation has had severe consequences in terms of health, financial crises, and education.

Merchant and Lurie^3^ conducted study in which they examined the importance of social media as a critical tool in managing the ongoing epidemic and the evolving characteristics of disaster preparedness and response. Doulamis et al.^4^ categorized Twitter data as per the tasks, event kinds, and the content orientation of tweets. Farzindar and Khreich^5^ conducted a similar study in which they examined event detection in Twitter data in terms of event types, characteristics, detection methods. Saeed et al.^6^ categorized existing methods for detecting Twitter events, highlighted their shortcomings, and rectified the deficiencies of existing approaches. Machine learning and hybrid method have been used to evaluate the implications of Covid-19 on socioeconomic condition using Twitter data. The previous work contribution shows in Table 1.

Twitter platform for collecting community opinion during the current Covid-19 outbreak. Twitter has an advantage over specific other social media platforms in that it provides immediate access to concise, real-time information and networks of comparable topics via #hashtags. Analyzing public responses and sentiment during the early stages of a pandemic in places with social inequalities might help inform future public health guidelines in similar conditions. We have collected the Covid-19-related data from the Twitter platform in our work. We extract Twitter data related to current challenges and discuss the prevalent public opinion around Covid-19. The findings of this research could help governments around the world design effective public health responses. Depending on local health infrastructure, economic position, demography, and varied regional circumstances, people expressed a range of perspectives, opinions, and feelings towards identical Covid-19 guidelines. During the various stages of covid-19 wave of the Coronavirus pandemic in 2021, the Indian government emphasized different public health guidelines, including “wash your hands,” “stay home,” “maintain a safe distance from other people,” “use the mask,” and “avoid coughing or sneezing in a public place.” Given the severity of the virus and the high mortality rate observed during the second wave in India, it was critical to stress the need of covid-19 appropriate behavior.

### Research motivation

People are being pushed out of public situations, and most of the coronavirus discussions take place on social media platforms like Twitter. So we can use Twitter data to mitigate and control the Covid-19 pandemic. The study aims to monitor public opinion to determine how their prospects, view, and opinions change with time in the country during a crisis. Analyzing a tremendous amount of community information can assist governments in analyzing public opinion over a period for making public health policy or applying safety guidelines. Opinion analysis finds out what the community is thinking about a subject. With the rise of coronavirus in late 2019 and its spread in early 2020, we will analyze how people think Covid-19 will affect over a period that has been a difficult situation by this disease.

### Problem statement

Social media platforms contain a tremendous amount of public opinion information to analyze and a decision-making system. So we need to examine public opinions around the Covid-19 situation, which helps us to analyze individual perceptions and make future policies. Previously, many countries have imposed lockdown as a solution to the covid-19.

The purpose of this work is to determine the Covid-19-related subjects and public opinion over the period of time stated on Twitter.

Research questions covered in this article are:

- How to automatically determine public perceptions stated on Twitter as a result of Covid-19?
- What are the most common challenges discussed while expressing their opinions about Covid-19 on Twitter?
- How to determine the Covid-19-related subjects and public opinion over the period of time stated on Twitter?

### Research contribution

The paper presents Twitter data as a source for opinion analysis. It is used to gauge public opinion and, more specifically, to track the subject associated with Covid-19. Also offers a systematic technique for analyzing sentiment classification for related variations in crisis scenarios, and association concerning Twitter data analysis. In this study, we proposed a deep transformer learning intelligence model to determine the social community opinion about Covid-19 in situations across the country. The proposed novel model would combine a transformer-based deep learning ensemble approach.

The contribution of this research work as follows :

- Sentiment analysis is used to examine public opinion toward Covid-19 related subject using Twitter data.
- The proposed method identifies public opinion in real-time and discusses the prevalent public dialogue around Covid-19.
- The proposed work classifies sentiments about Covid-19 related subjects over a period of time.
- The findings of this research could help governments around the world design effective public health decisions.

The remaining paper is organized as follows: Section “Related work”, discuss the related work. Section “Proposed method”, Proposed method. Section “Results analysis”, Results analysis. Section “Conclusion”, Conclusion.

## Related work

The scientific community interest in sentiment analysis is increasing because it facilitates decision-making for a variety of applications that rely on community opinion. As a result, previous studies various automated techniques for sentiment analysis ^7^. In investigated customized methods to identify constructs such as dominant behavior in electronic chats have and have demonstrated the potential to enhance analysis by expediting automated sentiment categorization utilizing NLP techniques ^8^. Covid-19 microblog texts can be analyzed in many ways, such as topics, market liquidity, events, and terms. Basiri et al.^9^ study investigated how Twitter users in eight countries felt about Covid-19. This work also has a new Covid-19 Twitter dataset that has taken over four months. Chakraborty et al.^10^ suggested a connection between people infected with Covid-19 and how many people died from Covid-19.

**Table 1:** Comparative analysis of different methods.

Reference	Methods	Techniques applied	Dataset	Task	Limitation
Barkur et al.^11^	Word frequency	Only WordCloud was used for the analysis, using the software R	Covid-19 Twitter	To investigated Indian public opinion following the government ordered lockdown	Depends only word frequency count only
Samuel et al.^12^	Machine learning	Classifiers such as naive bayes and logistic regression has been used	Covid-19 Twitter	In the work assessed the sentiment based on single keyword monitoring focused solely on the dread of people in the United States	Method depends Word frequency count and no context relation considered
Hamzah et al.^13^	Lexicon polarity	Predictive modeling of susceptible exposed- infectious recovered	Corona tracker website	To track the economic and health impacts on people as described on the corona tracker website	Polarity related to covid-19 keyword are neutral in majority of case
Abd-Alrazaq et al.^14^	Machine learning	Unigrams and bigrams was used to evaluate tweets, while dirichlet allocation was used topic modeling	Covid-19 Twitter	To derive a precise concept by analyzed the major subjects tweeted by netizens regarding the Covid-19 pandemic	Method not considered the context relation
lwin et al.^15^	Lexicon based	Lexicon based model analyzed the expression of various emotions in Covid-19	Covid-19 Twitter	Examined global trends in the expression of diverse emotions during Covid-19 pandemic	The situation of the Covid-19 case depends on the time and country
Raamkumar et al.^16^	Lexicon based	Recognize public health authorities communication methods for measuring public opinion and answers on social media	Facebook	The purposed of this study is to analyze public health authorities outreach activities to the public on Covid-19	Facebook data not related to Covid-19 context
Liu et al.^17^	Hybrid method	Categorizing contextual awareness through social via social media during Covid-19 pandemic	Covid-19 Twitter	Investigating the effects of Covid-19 on people mental health to aid policy and provide services to infected communities	These were largely concerns about health care
Satu et al.^18^	Machine learning	Suggested classification, clustered based approach examined subjects relevant to Covid-19	Covid-19 Twitter	Classify and examined sentiment relevant to Covid-19 as per topic	Very less tweets were determined from a single country
Wang et al.^19^	Machine learning	Covid-19 requires authorities and stake- holders to communicate about risks and crises	Covid-19 Twitter	Study evaluated the players risk and crisis communication on Twitter	Depended on the time and country
Su et al.^20^	Machine learning	LDA-Topic modeling was utilized to identify and track persistent difficulties	Covid-19 Twitter	Investigated to classify tweets according to country socio- economic condition	Topic depended only considered
Basiri et al.^9^	Deep learning	Fusion-based deep learning model	Covid-19 Twitter	Opinion analyzed on social media for regulating, and eradicating the condition	Method not covered opinion features
Proposed work	Transfer + Deep learning	BERT model for domain knowledge adoption and Bi-LSTM ensemble method	Covid-19 Twitter	Opinion analysis on Covid-19 related tweets over the period of time for the public opinion analysis system	

Recent research on Covid-19 has concentrated on the automatic recognition of tweets. Prabhakar et al.^21^ conducted a Covid-19 feature selection study that generated the frequently used tag used in Covid-19 tweets. Additionally also used the sentiment lexicon to determine the sentiments. In the work of^22^ ascertain the social and economic patterns associated with the Covid-19 outbreak in Pakistan. Huang et al.^23^ studied a total of 53k tweets from Saudi residents about Covid-19 and discovered that positive tweets outnumber negative tweets for nearly all of the metrics. They discovered that the processes associated with religious activity elicited the highest positive sentiment. They discovered that Saudi Twitter users support infection control efforts in the fight against Covid-19, and that this positive attitude among Saudi people contributes to the Saudi government overall trust. According to them, religious beliefs may be critical in preparing believers for pandemics. They gathered several tweets about the Saudi government various actions. After the news of the Grand Mosque’s closure, they analyzed 9924 tweets and discovered that 76.72% of them were positive. They also gathered tweets for the university closures, shopping mall, park, and restaurant closures, sports competition suspension hashtags, congregational and lastly, nationwide curfew restrictions. Depoux et al. ^24^ has shown that panic caused by people posting on social media spreads quicker than Covid-19. As a result, specialists and relevant authorities must notice and act to such rumors, attitudes, and public conduct as quickly as feasible^25^.

Users recalling side effects and starting to recollecting their previous infection with Covid-19 have been classified into various categories. The users could not be tested to confirm their concerns^26^. Because previous epidemics have been more modest, a recent investigation discovered a few studies that employed sentiment analysis to detect the presence of pandemics. Studies that assist authorities in appreciating human behavior may aid authorities in managing a crisis. Nowadays, social media is a primary source of news, and study that helps us understand human behavior may aid authorities in resolving a crisis Covid-19 ^27^. Rao et al.^28^ developed a method for doing efficient personnel screening, traveling history and general manifestations. The data acquired thus far may aid in initial screening and early detection of people who are Covid-19 positive. Data sets can be collected and enhanced using an artificial intelligence model, which can then be used to assess and categorize individuals who may be coronavirus positive ^29^.

Text categorization is a prominent field of research in NLP since it involves associating a given text sequence with predetermined categories. Numerous prior research has used government neural network models, and convolution neural network (CNN) model, to learn text presentation for categorization^30^. Recurrent neural network (RNN) and attention models outperform alternative statistical methods. Pre-trained word vectors across an extensive unsupervised document collection are frequently used as features of sequences in these studies, which are frequently trained using the word2vec ^31^ or the GloVe technique ^32^, which is based on the idea that words with similar meanings appear in similar contexts. So classifying the collection twitter data as per literature and statistics abound, and maintaining data up to date is challenging. In the work of^33^ integrates active learning into a system for sentiment analysis that also combines the most popular collectable methodologies. Souri et al.^34,35^ examined a user relationship management strategy in terms of the relationship between human behavior and social systems. The presented work in a formal framework that integrates the behavioral demonstrating method. Pashazadeh et al.^36^ systematic examined of the state-of-the-art mechanisms behind big data in healthcare applications.

Deep learning algorithms play an essential role in analyzing and predicting large epidemic data patterns and the early detection and exploitation of coronavirus outbreaks^37^. To evaluate the predictions with a positive, and negative opinion, the gated RNN and LSTM have presented the gated recurrent neural network ^38^. Deep learning has improved the performance of neural network architectures such as RNN, LSTM, and CNN ^39^ in solving a variety of NLP tasks such as text categorization, language processing, and machine translation ^40^. Text categorization, language modeling, machine translation, and other NLP activities are all sequence modeling challenges. Traditional machine learning methods and neural networks cannot grasp the text’s sequential information. A researcher began to employ RNN and LSTM due to their ability to model sequential data present in the text. In^41^ contributed attempting to address this issue by developing a sentiment-aware lexicon using data from many domains.

The lack of large labeled text datasets could be one of the fundamental causes for this poor progress. The majority of labeled text datasets are insufficient for training deep neural networks, as these networks contain many parameters, and training them on short datasets will result in overfitting ^42^. A deep learning model like this is referred to as a pre-trained model ^43^. As a result, rather than constructing a model from scratch, it is preferable to use a pre-trained model as a starting point to address a problem ^44^. One significant issue is that RNN cannot be parallelized because they only accept one input at a time. In the instance of a text sequence, an RNN or LSTM would take one token at a time as input.

Recently, despite the fact that BERT has produced some incredible results in various NLP applications and clearly surpasses most feature-based representation approaches ^45^, such as word2vec, GloVe full potential has yet to be realized ^46^. Additionally, the most common and best-performing solutions are more complicated language models. The Spatio-temporal model ^47^ is used to illustrate the dengue survival mechanism. The model is based on linear regression and is used to make predictions using tweet datasets. In the work  ^48^ of provides a concise overview of how NLP is beneficial for Twitter data analytics, used in research and public health analysis. Additionally, it has aided in deciphering social networks, public health messages, and the propagation of forecasts ^49^. Previous studies have demonstrated that pandemics and disease outbreaks can be contained if relevant experts consider publicly available data ^50^. Additionally, tracking twitter data has been used to study prior epidemics, do crisis situational analysis, and conduct tracking ^51^.

The strength of BERT is that the same pretrained model can be utilized in a variety of applications with excellent outcomes by simply adding one additional output layer. This means that it is capable of extracting critical information from input text that can be used to address a range of problems. Given that the model architecture is identical to that of the transformer encoder, we will concentrate on how it is trained and used for various purposes. BERT corpus is built of the Books Corpus(800 million words) and the English Wikipedia(2,500 M words). BERT comes in various flavors, including BioBERT ^52^, sciBERT ^53^, and others trained on domain-specific corpora. Pretraining model with such a large volume of material, it may learn the English language and develop the ability to extract meaningful information from the text to handle a variety of problems.

## Proposed method

**Figure 1:**
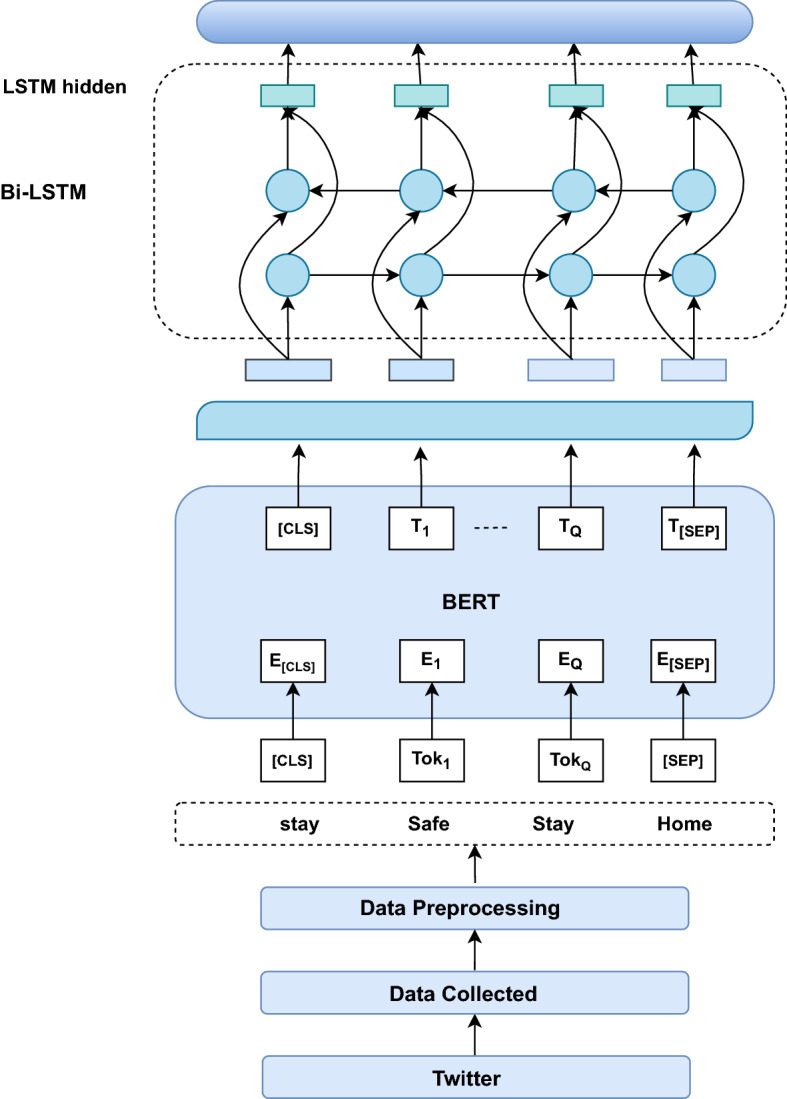
The architecture of BERT-Bi-LSTM Ensemble model for opinion analysis.

The proposed Bi-LSTM-BERT Ensemble approach consists of two stages. In the first stage, the BERT model gains the domain knowledge with Covid-19 data and fine-tunes Sentiment140 dataset. The second stage is the Bi-LSTM model, which is used to process the data in a bi-directional way to process the data to classify the sentiment. Finally, the ensemble model combines both models to classify the sentiment category into positive and negative categories. The goal of the approach is to categorize user opinions of tweets into two categories: positive sentiment or negative sentiment. It also analyzes opinions expressed in terms of various aspects at different times. The proposed method workflow is shown in Fig. 1.

### Task definition

Suppose Tweet contain sentence S and sequence of word w in sentence such as $S = [w_1, w_2,\dots ,w_n ]$ having ’n’ words and several aspect as mentioned in Table 3. The objective of the proposed work is to classify sentiment into positive and negative categories. For example, “Together, we can win the battle against COVID-19.” which is supposed to produce positive outputs. In the second case “Bullshit !! Our country is the worst in the world for the pandemic because of you, not China. Stop blaming everyone else & trying to defect blame. The state of our country is your fault & yours alone. RESIGN” which shows negative intent. In third case “maybe being stupid is a pre-existing condition that makes you susceptible to the corona virus?” which shows negative intent. In the fourth case “Covid-19 is everyone’s fight. I have covered major disasters, but nothing like this before” which shows negative intent. For better understanding, we have given some examples in Table 2.

**Table 2:** Some sample example from Covid-19 data collection.

Sr. No.	Sample example	Category
1.	Stay safe stay home.	Neutral
2.	We can fight against COVID-19 and beat him.	Positive
3.	It is vital to follow the guideline to prevent the spread of COVID-19.	Positive
4.	Can Zinc medicine treat corona, Don’t believe it.	Negative
5.	Covid-19 is everyone’s fight. but nothing like this before happened.	Negative

### Data collection

The data collection stage consists of English-language Twitter post (tweets) shared by users between 1st March, 2021 to 1st January, 2022. We used the keyword (top frequent hashtag) search tagging word, phrase mentioned in Table 3. The Twitter standard search API returns specific tweet properties for each tweet, including the tweet unique identified user ID, the tweet time and content, and geo-graphic coordinates location (including latitude and longitude) denoting the tweet’s boundary ( https://developer.twitter.com/en/docs/twitter-api/v1/tweets/filter-realtime). Twitter does not identify the precise location from which tweets were sent in-country for privacy concerns. In our data, all the tweets are collected in India (https://twitter.com). We crawled tweets from the Twitter platform, including Covid-19 related top frequent associated tags with tweets. Our study covered several issues, including covid-19, lockdown, vaccination, health, quarantine, safety, policy, and guidelines. Finally, we have combined all the datasets to analyze the public perception corresponding to associated tags to classify them into positive and negative category. The data set (tweets) has been obtained using Tweepy, an official Python Twitter API library, and the data set (tweets) has been obtained (https://github.com/MayurWankhade/Sentiment-Classification-Task). The selection criteria are based on the tweet are based on the associated tag as mentioned in Table 3.

**Table 3:** Top frequent tag used in collecting data from twitter.

Aspect	Related hashtag (#)
Covid-19	COVID19, corona, covid, coronavirusindia corona, coronavirus, IndiaFightsCorona,coronavirus, virus, Covid-19
Vaccination	firstdose, vaccine, CoWin, seconddose COVAXIN, Covishield, sputnikvaccine, vaccineregistration, Modernavaccine, Novavaxvaccine, COVID-19vaccine
Lockdown	covidprotocol, adversary, quarantine-life, quarantine, stayhome, stayhomestaysafe, MentalHealthAwarenes, coronawarrior, SocialDistancing, StayHome, StayAtHome, SocialDistanacing, WorkFromHome, washyourhands, BackToWork, SayNoToMasks, lockdown2021, CoronaLockdown, StayAtHomeAndStaySafe, lockdownguideline

### Data preprocessing

Data preprocessing is one of the most effective ways to extract stated emotions from unstructured texts by converting the information into a structured manner. We performed the data preprocessing task to remove irrelevant data in tweets required for effective categorization; it is necessary to remove unnecessary data to reduce the dimension of data which is helpful to improve the model performance. The Python library package has been used to collect geographic location tweets for the key tagging phrases and timing period. Only English tweets are considered for further data analysis. The data collected from tweets are cleaned, removing the unnecessary symbols commonly included in tweets. The “re” python package cleans symbols like # , @, URL, RT, number values, and punctuation marks. Finally, data is stored in a structured format and removes irrelevant and duplicate rows of similar tweets. The preprocessing stage includes remove the stop-words, URL, # and number also consider replacing negation mentioned and repeated character removal, which effectively improves sentiment classification accuracy. The statistics of collected data are shown in Table 4.

**Table 4:** Statistics of the collected datasets.

Dataset	Total number of tweet	Positive word count	Negative word count	Neutral word count	Average word length
Covid-19	465,639	245,983	223,441	345,350	15.07
Vaccination	122,521	61,109	4242	5450	11.02
Lockdown	380,359	180,311	245,780	105,790	16.38

### BERT-Bi-LSTM ensemble model

The proposed approach consists of two stages. In the first stage, the BERT model which is used to gains the domain knowledge of Covid-19 data and fine-tunes with Sentiment140 dataset. Because Covid-19 dataset most of the words are new to understands for machine so to gain the domain specific knowledge BERT model used in approach. The second stage is the Bi-LSTM model, which is used to process the data in a bi-directional way to process the data to classify the sentiment.

The proposed BERT-Bi-LSTM Ensemble model has used to classify the public opinion into positive and negative category. Sentiment analysis is one of the most effective ways to analyze user opinion. To make opinion sense of the massive tweets shared on Twitter platforms at various times. The sentiment characteristics of text are determined by the sentiment polarities of its many aspects, such as positive and negative intent words and emoticons. Sentiment features consist of positive and negative intent words or phrases. Additionally, we consulted sentiment strength when developing our vocabulary of emotional terms, but we are currently extending our own (https://github.com/MayurWankhade/Sentiment-Classification-Task). The emoticons features are positive, negative, and neutral. Features relating to hashtags include the total number of positive and negative hashtags. The number of positive and negative slang terms is one of the characteristics of slang words.

#### BERT domain knowledge adoption

We present the BERT attention mechanism contains two essential modules: the Covid-19 domain attention and sentiment modules. The domain attention mechanism module gains the domain representation invokes an domain-related features. BERT is intended to pre-train deep bidirectional representations from an unlabeled text by simultaneously conditioning both the left and right contexts. As a result, by adding a single additional output layer to the pre-trained BERT model, for a wide variety of NLP applications can be generated ^54^. Bidirectional means that the NLP BERT framework acquires knowledge about a word’s in bi-directional way. After pre-processing data training step, acquiring data train from the BERT model and output of BERT passes to Bi-LSTM model. To simulate the logarithm of probabilities, we employ a fully connected linear layer.

1 $$\begin{aligned} L_p = W^Tx + b, \end{aligned}$$

The *i*th element of $L_p$ is the log-likelihood of the *i*th tag, indicate the word embedding. Input of sequence taken as $X = (x_1,x_2,\ldots ,x_n)$ and the output label obtained with fine tune BERT as $Y = (y_1,y_2,\ldots ,y_n)$. We obtain the predicated tags by selecting the tag with the highest probability from a sequence of word embedding.

2 $$\begin{aligned} S (X, y) = \sum _{t=1}^n A_t y_t + \sum _{t=0}^n B_{y_t,y_{t+1}} \end{aligned}$$

A softmax all-inclusive conceivable tag sequence produces the following probability for the sequence y:

3 $$\begin{aligned} p (y/X) = \frac{e^{S(x,y)}}{\sum _{\bar{y} \epsilon Y_X} e^{S(x, \bar{y}) } } \end{aligned}$$

We enhanced the log-probability of the proper tag sequence during training:

4 $$\begin{aligned} log(p(y|X)) = s (X, y) - log \Big ( \sum _{\bar{y} \epsilon Y_X} e^{s(X, \bar{y})} \Big ) \end{aligned}$$

where $Y_X$ denotes all possible unique tag sequences for phrase X. During decoding, we forecast the output sequence with the highest score given by

5 $$\begin{aligned} y^*= argmax_{\bar{y} \epsilon Y_X} S(X,\bar{y}) \end{aligned}$$

Twitter data does not include a sentiment label, so it is challenging to train the model. In the proposed model for data training and fine-tuning performed with Stanford Sentiment140 dataset ^55^ to solve the unlabeled data issue, sentiment140 data consist of 1600 million tweets with positive and negative sentiment labels. We randomly picked 75% of the tweets used for training the models and 25% for validation during the testing process.

**Reason for selecting BERT model**: BERT can produce multiple words embedding for a single word, each of which reflects the context of the word as it appears in a sentence. Transformer fundamental idea has been to integrate the benefits of CNN and RNN in a unique design based on the attention mechanism. Transformer architecture provides parallelism by paying attention to the recurrence sequence and simultaneously encoding the position of each item in the sequence. As a result, a compatible model with a significantly reduced training time is obtained.

#### Bi-LSTM attention mechanism

LSTM ^56^, has been utilized as a more advanced form to overcome RNN limitations by introducing hidden layer units known as memory cells. Memory cells are self-contained units that store the temporal network state and are controlled by input, output and forget gates. The input gate function regulates the flow of memory cell input and output gate into the remainder of the network. The activation level determines the information stored in memory. If the input unit has a high activation level, the information is stored in a memory cell. Additionally, if the output unit has a high activation level, it will transmit information to the next neuron. Else, high-weighted input data is stored in a memory cell. Bi-LSTM attention mechanism consists of forward and backward steps which used to identify the true meaning of the sentence. The forward step notations are the activation vectors of $i_t$ denote input gate as per Eq. (6), $f_t$ denote forget gate as per Eq. (7), $c_t$ denote output gate as per Eq. (8), and $c_t$ denote cell gate as per Eq. (9), and $\sigma$ is the sigmoid function. We use LSTM networks layers, which are utilized in sequence labeling tasks and produce significant results. We use the following method of implementation:

6 $$\begin{aligned} i_t&= \sigma _g(W_i x_t + W_{hi} h_{(t-1)} + b_{hi}) \end{aligned}$$

7 $$\begin{aligned} f_t &= \sigma _g(W_f x_t + W_{hf} h_{(t-1)} + b_{hf} ) \end{aligned}$$

8 $$\begin{aligned} o_t &= \sigma _g(W_o x_t + W_{ho} h_{(t-1)} + b_{ho}) \end{aligned}$$

9 $$\begin{aligned} c_t &= f_t c_{(t - 1)} + i_t \odot \mathrm{tanh}(W_c x_t + W_{hc} h_{(t - 1)}) + b_{hc} ) \end{aligned}$$

10 $$\begin{aligned} h_t &= o_t \odot \mathrm{tanh}(c_t ) \end{aligned}$$

The backward step notations are the activation vectors of $\tilde{i_t}$ denote input gate as per Eq. (11), $\tilde{f_t}$ denote forget gate as per Eq. (12), $\tilde{o_t}$ denote output gate as per Eq. (13), and $\tilde{c_t}$ denote cell gate as per Eq. (14), and $\sigma$ is the sigmoid function.

11 $$\begin{aligned} \tilde{i_t} &= \sigma (W_i x_{t} + W_{hi} h_{(t + 1)} + b_{hi}) \end{aligned}$$

12 $$\begin{aligned} \tilde{f_t}&= \sigma (W_f w_t + W_{hf} h_{(t + 1)} + b_{hf} ) \end{aligned}$$

13 $$\begin{aligned} \tilde{o_t} &= \sigma _g(W_f x_t + W_{ho} h_{(t+1)} + b_{ho} ) \end{aligned}$$

14 $$\begin{aligned} \tilde{c_t} &= f_t c_{(t - 1)} + i_t \odot \mathrm{tanh}(W_c x_t + W_{hc} h_{(t + 1)}) + b_{hc} ) \end{aligned}$$

15 $$\begin{aligned} \tilde{h_t} &= o_t \odot \mathrm{tanh}(c_t ) \end{aligned}$$

Where, W indicate the weight matrices, b indicate bias vector parameters, $x_t$ indicate the input variable at time *t*, $h_{t-1}$ is the hidden state of the layer at time $t-1$, $h_t$ is the hidden state at time t, $c_t$ is the cell state at time t. We have adopted Bi-LSTM attention mechanism from^57^ and ensemble model construct the domain representation using a Bi-LSTM network in the domain attention mechanism module. The $f_{Bi-LSTM}(\dot{)}$ used to represent the word embedding process, we can formulate this process as

16 $$\begin{aligned} h_d = f_{Bi-LSTM}(x_i^j) = f_{Bi-LSTM}\big (wv_1,wv_2,\dots ,wv_n)\big ) \end{aligned}$$

Where, $wv_i$ represents the word vector for the $i{th}$ word in the sentence. Specifically, $h_d$ is the sum of the outputs of a forward LSTM network as per Eq. (10) and a backward LSTM network as per Eq. (15) as follows

17 $$\begin{aligned} h_d = \overrightarrow{h_t} \oplus \overleftarrow{\tilde{h_t}} \end{aligned}$$

Where as $\oplus$ indicate the concatenation and function $f_{Bi-LSTM}()$ transform the text embedding. The domain knowledge representation is fed into softmax classifier to gain the domain information.

18 $$\begin{aligned} h_d &= f_{Bi-LSTM}(x_i^j) + f_{Bi-LSTM}(x_i^j) \end{aligned}$$

19 $$\begin{aligned} y_i^{attention} &= f(W^{attention} (h_d + h_i^s ) + b^{attention}) \end{aligned}$$

Where $W^{attention}$ and $b^{attention}$ are parameters for attention. Then, all attention weights are fed into a softmax layer to generate probabilistic attention weights. The last stage representation for classification tasks $h^{s}$ is the weighted combination of all memory formation which is,

20 $$\begin{aligned} \alpha _i &= \frac{exp(y_i)}{\sum _{i=1}^n exp(y_i)} \end{aligned}$$

21 $$\begin{aligned} h_s &= \sum _{i=1}^n \alpha _i h_i^s \end{aligned}$$

The weighted vector $h_{s}$ can be viewed as a hidden layer of a text for sentiment classification and fully-connected layer and a softmax layer in predicting the sentiment labels of texts. As per Eqs. (21) and (19) mapping between the acquired hidden layer and its sentiment label and s is the predicted sentiment label.

## Result analysis

The experiments are executed using NVIDIA Tesla V100 SXM3 32 GB GPU. The hyper-parameter setting as follows dimensions of hidden size BiLSTM is = 300, dropout rate = 0.2, learning parameter = 0.001, regularization weight = 0.001, activation function as sigmoid, BERT model parameter are consists of: number of layers = 12, hidden size h = 768, self-attention heads A = 12, total parameters= 110M. We have validated the model by modifying several hyper parameters and provided the optimal result. We have manually checked parameters that are perfectly suited to the proposed method. We have performed the ten cross-validation (CV) and average accuracy results considered in the proposed work.

**Figure 2:**
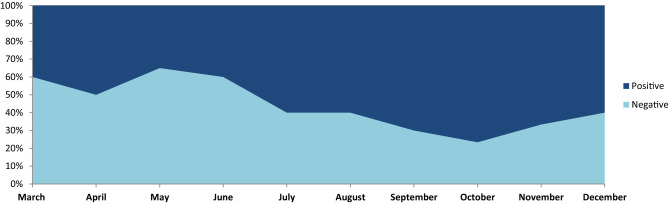
The overall public opinion discussed during Covid-19 pandemic condition month-wise result analysis.

**Figure 3:**
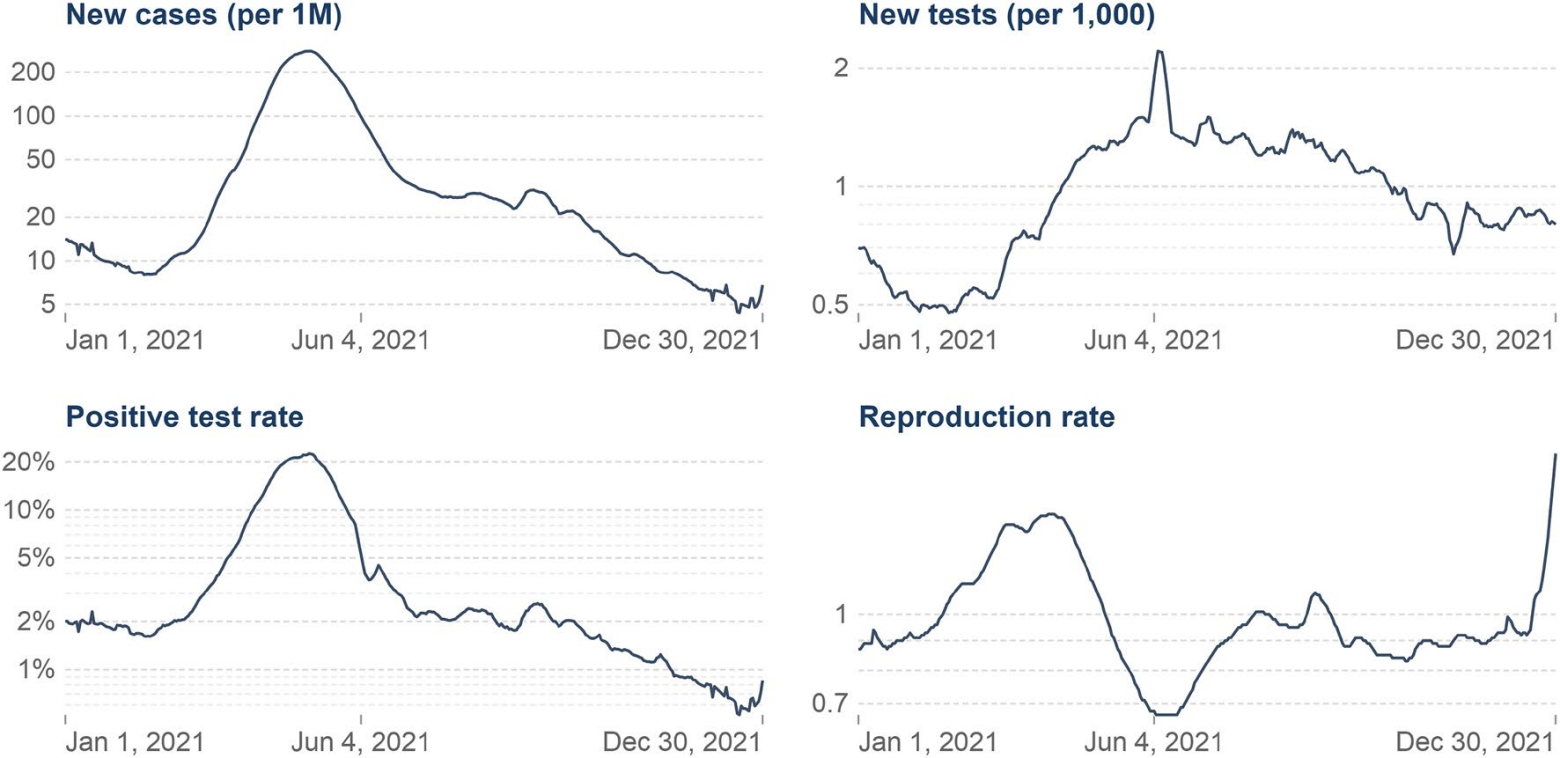
Graphical visualization of Covid-19 cases, the report contains a record of new case, new taste case, positive rate, reproduction rate, and over the period.

The proposed BERT-Bi-LSTM ensemble method analyzes public opinion and categorizes it into positive and negative categories to find out the overall sentiment. We examined how country-specific(India) community opinions around the Covid-19 epidemic are evolving. A massive amount of public opinion analysis plays an essential role in measuring sentiment for government to make specific decisions. We extracted public opinion from Twitter posts at the country level(India) at different times in order to analyze how public opinion of the pandemic has evolved. The proposed method classified each tweet’s valence (positive or negative) based on its textual information emotion intensity. Additionally, a key aspect related to the Covid-19 is classified as aggregated sentiment across tweets. The distribution of opinions is found in Fig. 2 shows inconsistency across period (1st March, 2021 to 1st January, 2022) in the country (Fig. 3).

This paper presents a novel BERT-Bi-LSTM ensemble approach for sentiment analysis. In our work, we have used the CuDNN package to accelerate GPU computation. The computational time for the proposed BERT-BiLSTM ensemble approach for training the data has taken 11.73 s, whereas testing the data has taken 03.77 s. At the same time, to fine-tune domain knowledge, BERT has taken 6.07 s to gain sentiment domain information. The proposed method result analysis compare with baseline machine learning and deep learning method in Table 5.

In the proposed model, several variations of the result shown in Table 6 indicate the impact of each stage on classifying the sentiment. Since the Covid-19 dataset, most words are unique, and it is not used to classify sentiment. To solve these challenges in the proposed work, we have used the sentiment140 dataset to gain domain knowledge for finding categories. Also, we found that the result obtained after adding sentiment dictionary positive and negative features to the sentiment fine-tune (SFT) BERT model improved the classification accuracy. Table 5 experiment results show the impact of dictionaries in classification; the F1 score rises to 86.13% (compared to 73.48%). Adding sentiment features can also significantly boost performance. When the Bi-LSTM model with hidden layer attention embedding is used for sentiment features, the F1 score can rise to 86.13%, respectively (compared to 75.14% ). Furthermore, when dictionary and emotion characteristics are combined, the model yields an F1 score of 86.13% and 85.78% for positive and negative categories. Also, the most common aspects discussed during the pandemic and overall public opinion are shown in Table 7.

**Table 5:** Proposed model comparison with baseline method on the Covid-19 dataset.

Model	Classifier	CV training	CV testing	Accuracy	Time taken (in second)
BoW	Naive bayes	68.141	58.147	61.365	12.36
	Support vector machine	82.451	70.027	72.895	16.25
	Random forest	74.956	95.360	62.698	123.22
	Logistic regression	66.254	76.589	71.587	17.26
N-gram	Naive bayes	70.640	65.107	66.160	14.09
	Support vector machine	84.051	73.065	74.290	18.20
	Random forest	76.956	95.360	65.018	133.08
	Logistic regression	73.057	74.049	72.527	21.23
word2vec	LSTM	75.026	70.568	73.897	27.14
GloVe	LSTM	76.714	72.358	74.106	30.47
BERT	Bi-LSTM	82.914	80.478	82.479	26.45
Proposed	BERT-Bi-LSTM ensemble	86.041	86.450	86.139	26.40

**Table 6:** The proposed model with several variations comparative result in analysis on the Covid-19 dataset.

Method	Positive			Negative		
	Precision	Recall	F-measure	Precision	Recall	F-measure
BERT	74.14	75.07	75.14	74.47	75.14	75.47
Bi-LSTM	72.41	73.48	73.45	74.09	75.01	74.89
SFT-BERT	78.04	78.58	78.65	75.09	76.02	76.14
BERT+LSTM	79.74	79.89	79.95	79.18	80.14	80.49
SFT-BERT+LSTM	83.47	84.75	85.66	83.14	84.01	84.17
SFT-BERT+Bi-LSTM	86.04	86.45	86.13	84.01	85.47	85.78

The results indicate in Fig. 6 shows the average strength of negative feelings reduced, and the intensity of positive sentiments grew between the beginning of the pandemic and the prospect of the country’s reopening. The various phase of data collecting occurred during an era of unequal lockdown measures, resulting in high scores for negative emotions and low values for pleasant emotions. The most challenging aspect discussed during the pandemic period, and the sentiment classification performance of various aspects are shown in Table 5.

**Table 7:** Aspect attention base sentiment classification using the proposed method.

Aspect	Positive			Negative		
	Precision	Recall	F-measure	Precision	Recall	F-measure
Covid-19	82.01	81.14	81.47	85.19	85.94	86.01
Lockdown	85.11	85.39	85.65	83.47	84.22	84.32
Vaccination	87.66	87.96	87.82	82.14	83.01	83.16
Health	85.14	85.36	85.47	85.77	85.89	85.44
Quartine	82.14	83.01	83.16	85.42	86.31	86.11
Safety	83.47	84.12	84.32	87.06	87.26	87.82
Policy	84.38	84.79	84.58	82.04	83.01	83.16
Guideline	85.47	85.89	85.45	85.14	85.36	85.37
Overall	86.04	86.45	86.13	84.01	85.47	85.78

**Figure 4:**
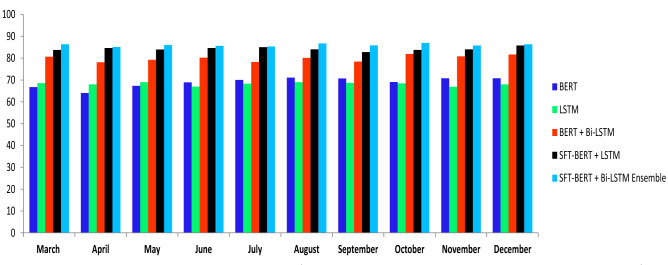
The proposed method comparative result analysis monthly reported during pandemic a period.

**Figure 5:**
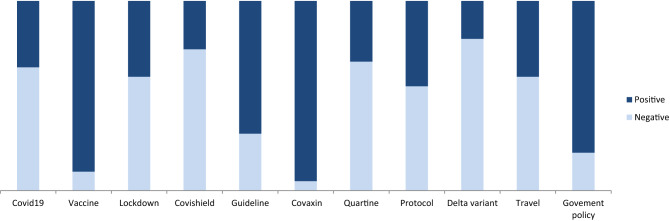
Overall public sentiment classification on various aspects discussed during the pandemic period.

Figure 3 shows the Covid-19 cases, the report contains a record of a new case, new taste case, positive rate, reproduction rate, and over the period. The proposed method result comparison shown in Fig. 4 indicate that result obtained are consistent for real-time public opinion analysis over a period. Depending on Covid-19 cases over the period and public opinion impact based on that time, Fig. 7 shows that public opinions are getting changed as per the covid-19 situation in the country (https://ourworldindata.org/covid-cases). It depends on the Covid-19 situation in the country. How the people lockdown aspect gets change over time are shown in Fig. 5. The people get affected due to Covid-19, new confirmed Covid-19 cases reported, and death cases reported over the period in India are shown in Fig. 9, whereas 1M indicates per million.

**Figure 6:**
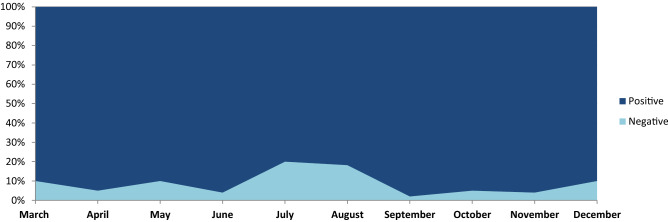
Public opinion analysis on vaccination discussed during the pandemic period.

**Figure 7:**
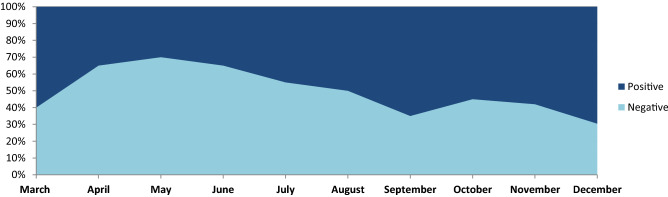
Public opinion analysis on lowdown discussed during the pandemic period.

**Figure 8:**
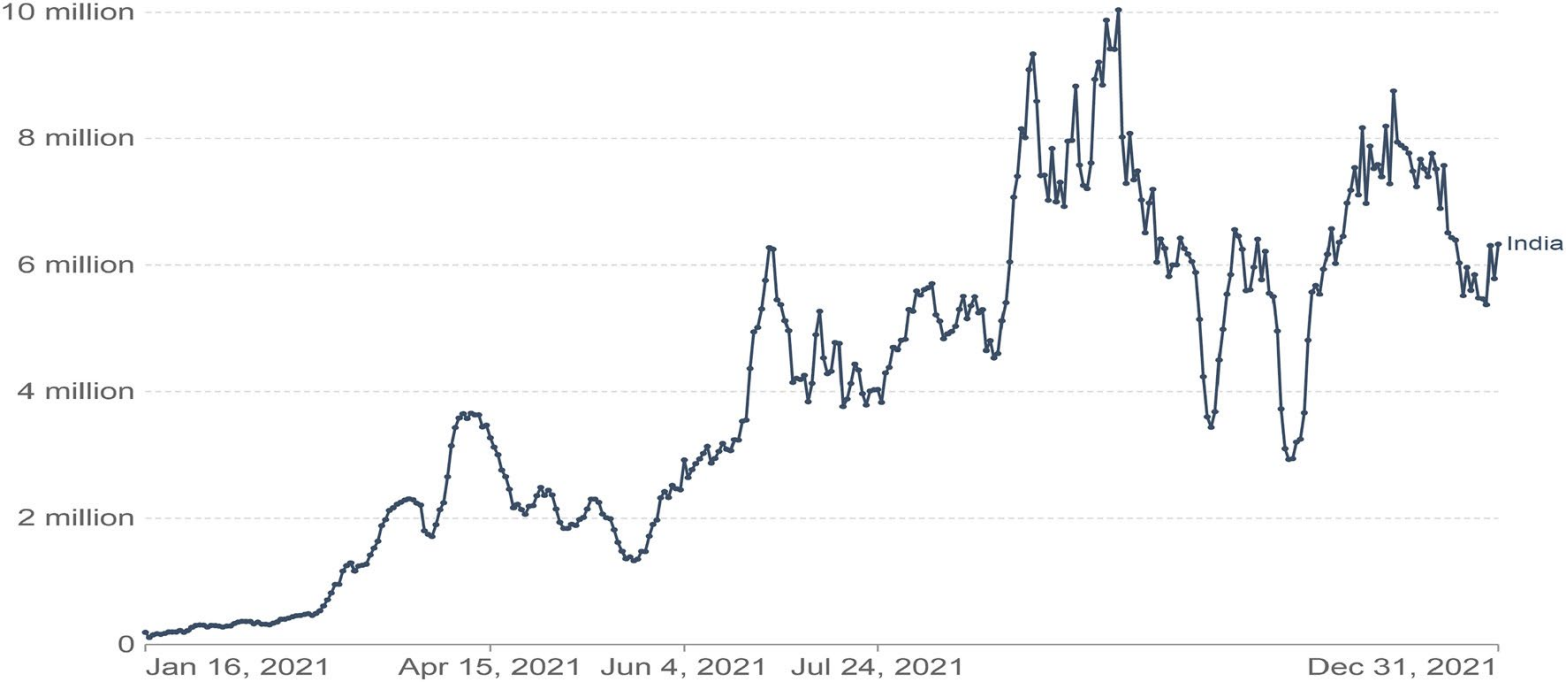
The number of people vaccinated reported over the period of time.

**Figure 9:**
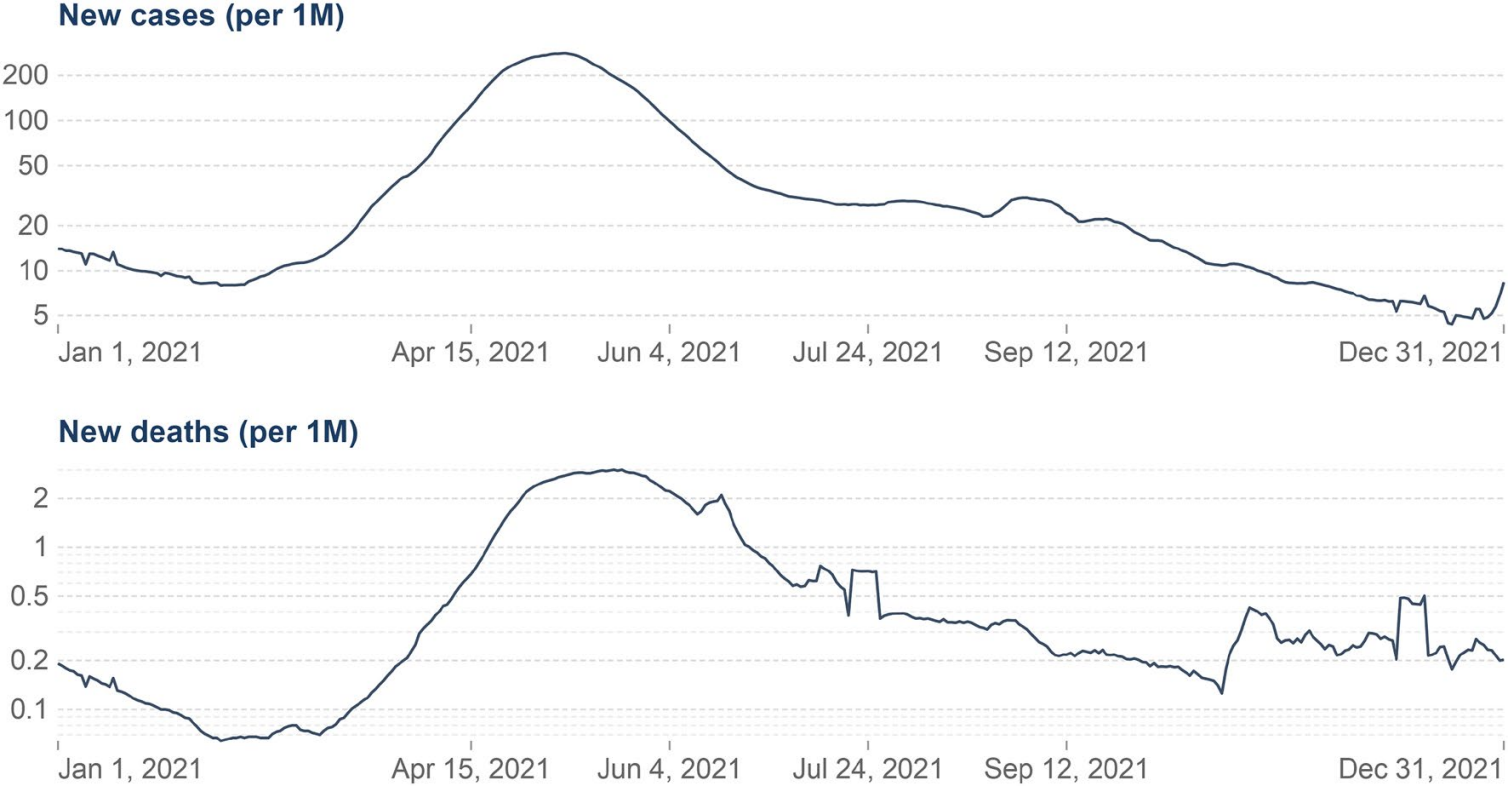
The ratio of new confirmed Covid-19 cases reported and death cases reported over the period.

The public opinion on Covid-19 as per the vaccination-related tag mentioned in Table 3 over Twitter indicates most users positive opinions and graphical visualization over the period shown in Fig. 6. The impact of vaccination indicates positive things towards public safety (Fig. 7). Figure 8 shows the number of people vaccinated reported over the period in India (https://www.mohfw.gov.in). The number of new cases reported and death cases reported over time in India is shown in Fig. 9.

For comparison, we employed the Bag of Words (BoW)^58^ and N-gram models^59^ Word2Vec and GloVe with various classifiers Naive bayes, Support vector machine, Random forest, and Logistic regression. Also, we have used a deep learning approach as LSTM and Bi-LSTM. Compared to the traditional machine learning approach, the deep learning approach demonstrated superior performance for the Covid-19 dataset; compared to baseline methods the proposed approach showed significantly superior performance due to its ability to capture out-of-vocabulary words effectively. The comparative result analysis shown in Table 5.

**Figure 10:**
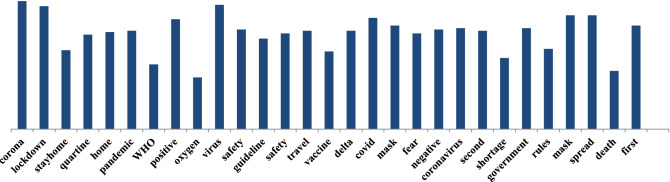
Top frequently used words which are most commonly discussed during the pandemic period.

Additionally, we perform in-depth social sentiment research via public opinion to ascertain the general population’s feelings. The most emerging topic identified by the top frequently mentioned in Fig. 10 is most commonly discussed during the pandemic. Also, most aspects discussed during the pandemic and overall public opinion are shown in Fig. 5. We found that negative opinion dominates public sentiment regarding these critical Covid-19 occurrences, a consistent pattern across countries. This can help governments and organizations learn more about their involvement with this disease to develop a better decision-making policy to help their citizens.

## Discussion

The proposed work classifies the sentiment into positive and negative categories, which is helpful for the decision-making system. Our findings are based on the opinions or feelings of people in India discussed on Twitter social media platform about Covid-19 related subjects. Twitter is an incredibly powerful and efficient platform for communicating the efficacy of the public is shown. Real-time epidemiological data is necessary for self-reporting capabilities and rapid assessment of pandemic scenarios. The Covid-19 dataset has vast scales of unavailable training data are due to the time and human resources required for manual labeling of training data. It is not possible to label such tremendous data. In the proposed approach data training and fine-tuning performed with stanford sentiment140 dataset to solve the unlabeled data issue.

The proposed approach to investigating content related to Covid-19 reveals that Twitter may be efficiently used to identify individual-level responses to infectious disease outbreaks. At the same time, it considers the effects of local socioeconomic resources and illness prevalence. Additionally, it established a socioeconomic difference and reactions to the current Covid-19 outbreak even in the country where disease cases are the most severe. Also, it provides a comprehensive study of public sentiment, including the overall state of public emotions, the change in public sentiment over time, and the emotions expressed in response to specific occurs. The COVID-19 pandemic threatens the physical and mental health of millions. This article evaluated how feelings and emotions about the pandemic evolved over the period of time. The empowered ct of lockdown Stanford ination measures the hope of a return to normal with an effective vaccination effort and a decline in incidence.We analyze the variations in opinion under lockdown regulations. When comparing impact of lockdown measures with respective synthetic control, we find some evidence to support the widespread belief that lockdown policies have high emotional consequences. Also, some significant point are discuss below

- Since the Covid-19 dataset, most of the words are unique does not include any sentiment label. To gain the domain knowledge and sentiment knowledge with sentiment140 corpus, which has extensive volume label data used to assign the label in our work. Sentiment fine-tuned BERT training on the Twitter Covid-19 dataset improves the performance of the proposed model.
- Adding Bi-LSTM hidden attention layers on top of the BERT model improves the performance of the proposed method.
- We use tweet locations to investigate the county-specific(data explicitly collected in India) geographic distributions of Covid-19 tweets.
- We summarize and reveal the aspect addressed on Twitter by tagging based attention with proposed models.

## Conclusion

We demonstrated the performance of the proposed model by comparing it with various versions of the BERT methods. We analyze social media, i.e., Twitter discourse regarding COVID-19, by utilizing information from tweets tagged with Covid-19 related topics and various challenges discussed during the pandemic, such as feelings, subjects, and emerging issues. We perform a real-time analysis of public opinion changes related to Covid-19. The proposed approach comprehension the changing nature of people’s opinions about the pandemic duration to be helpful for the government in making decisions. This study analysis of community-based pandemic reactions might reveal disparate discourses about social life such as mental health, daily routine, socio-economic disparity, and education. Such information can assist in initiatives such as public health crisis messaging and prioritizing the interests of the disproportionately affected people geographic.

## Data Availability

The datasets used analyzed during the current study are available from the corresponding author on reasonable request. The codes that were made or used in this study are eligible for inclusion author on reasonable request. Details information is available through requests from the authors.
